# Phenology, nectar dynamics and reproductive success of *Inga vera* (Leguminosae) in monospecific plantations and forest remnants in Atlantic forest: Dataset exploration

**DOI:** 10.1016/j.dib.2018.08.051

**Published:** 2018-08-24

**Authors:** Oswaldo Cruz-Neto, Jéssica Luiza Souza e Silva, Marcela Masie Woolley, Marcelo Tabarelli, Ariadna Valentina Lopes

**Affiliations:** a Departamento de Botânica, Universidade Federal de Pernambuco (UFPE), Recife, PE 50372-970, Brazil; b Programa de Pós-Graduação de Biologia Vegetal, Universidade Federal de Pernambuco (UFPE), Recife, PE 50372-970, Brazil

## Abstract

Distinct approaches have been adopted for tropical forest restoration in the last decades. The long-term viability of these restored forests and their capacity to provide the required services demand continuous evaluation to guide future initiatives. In addition, the resilience and maintenance of plant and animals communities in restored forests in tropical regions are closely related to the recovery of animal-mediated interactions. We provide, in this article, raw data related to the reproductive ecology of *Inga vera*, one of the most common tree species used in forest restoration initiatives in the Brazilian northeastern Atlantic forest. Specifically, we explore data on phenology, nectar dynamics and reproductive success of *I. vera* in natural and planted (monospecific) populations. This data article is related to the research article “*Pollination partial recovery across monospecific plantations of a native tree (Inga vera, Leguminosae) in the Atlantic forest: lessons for restoration*” (Cruz-Neto et al., 2018) [1].

**Specifications Table**

**Table:** 

Subject area	*Biology*
More specific subject area	*Plant reproductive ecology, pollination ecology, restoration ecology*
Type of data	*Table, text file and graphs*
How data was acquired	*Binoculars for phenological data; microsyringes (Hamilton Microliter*^*TM*^*10 and 25 μl) and a hand-held refractometer (Atago*® *0–50%*) *for nectar measurements; and microscopes for evaluating male reproductive success ofI. vera*
Data format	*Raw and filtered data*
Experimental factors	*Sampled flowers used in nectar experiments were previously bagged to prevent visits by pollinators. For the investigations related to the growth of pollen tubes, pistils of flowers were previously fixed in formalin–acetic acid–alcohol (FAA)*
Experimental features	*Many components of tree reproduction such as, synchronization of reproductive phenology, floral nectar secretion and consumption, fruit set, seed set and proportion of pistils with pollen tubes were compared among eight populations of I. vera distributed in four monospecific plantations and four forest remnants*
Data source location	*Usina Serra Grande, Ibateguara Municipality, Alagoas State, Brazil (8*^*o*^*58’50”S, 36*^*o*^*04’30”W).*
Data accessibility	*Data is with this article.*
Related research article	*Data presented in this brief is related to the study “Pollination partial recovery across monospecific plantations of a native tree (Inga vera, Leguminosae) in the Atlantic forest: lessons for restoration, Forest Ecol. Manag. 427 (2018) 383–391”*[1]

**Value of the data**

• Data of synchronization in reproductive phenophase may be useful to infer if planted populations of trees may supply food resource for pollinators and seed dispersers from surrounding forest remnants.
• Nectar secretion, availability and consumption may support inferences about resource use by pollinators in human modified landscapes.
• As a final variable of the process, fruit set per tree or population is a reliable measure of the efficiency of pollination in planted and natural populations of obligatory cross-pollinated tree species.
• These data are suitable to support decision-making aimed at recovering the pollination process in future forest restoration initiatives in tropical forests.

## 1 Data

**Fig. 1:**
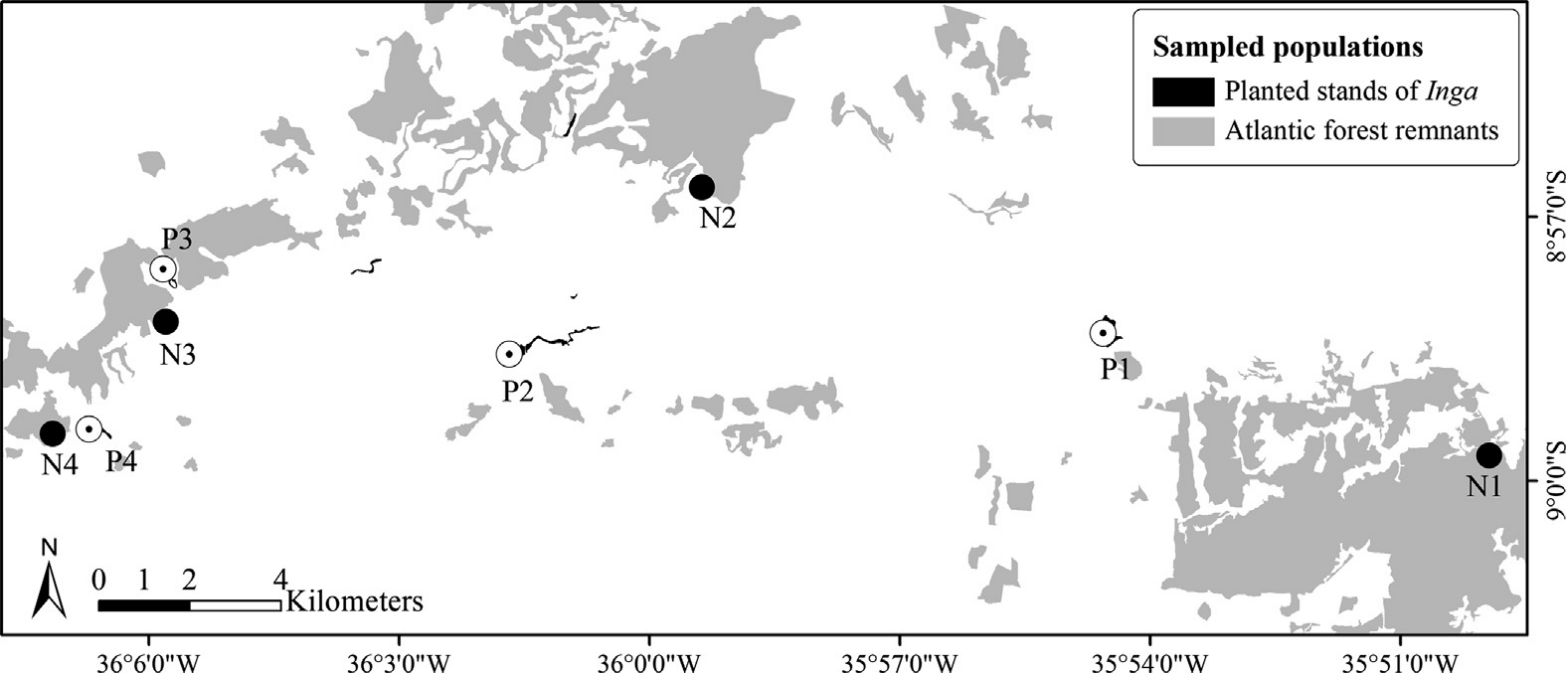
Description and location of the study area at the Brazilian northeastern Atlantic forest, Alagoas state. Map of study site highlighting remnants of Atlantic forest and reforested areas. Sampled populations of *Inga vera* in forest remnants (N1-N4) and reforested areas (monospecific plantations) (P1-P4) are highlighted in the map.

**Fig. 2:**
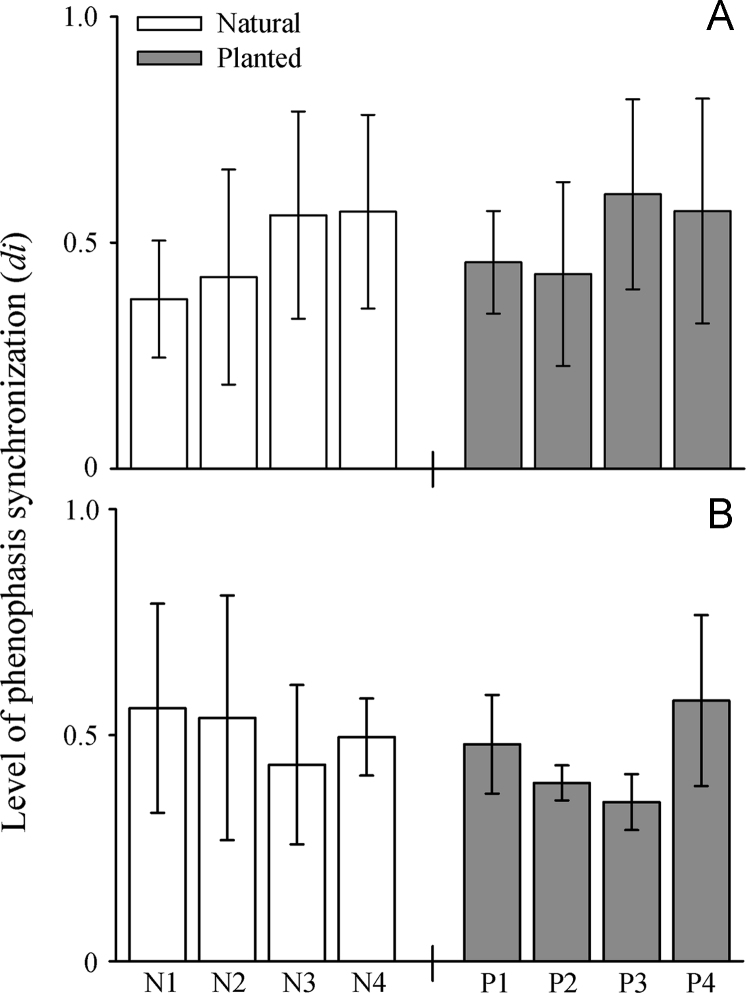
Flowering (A) and Fruiting (B) emission of *Inga vera* across natural and planted populations at the Brazilian northeastern Atlantic forest, Alagoas state.

**Fig. 3:**
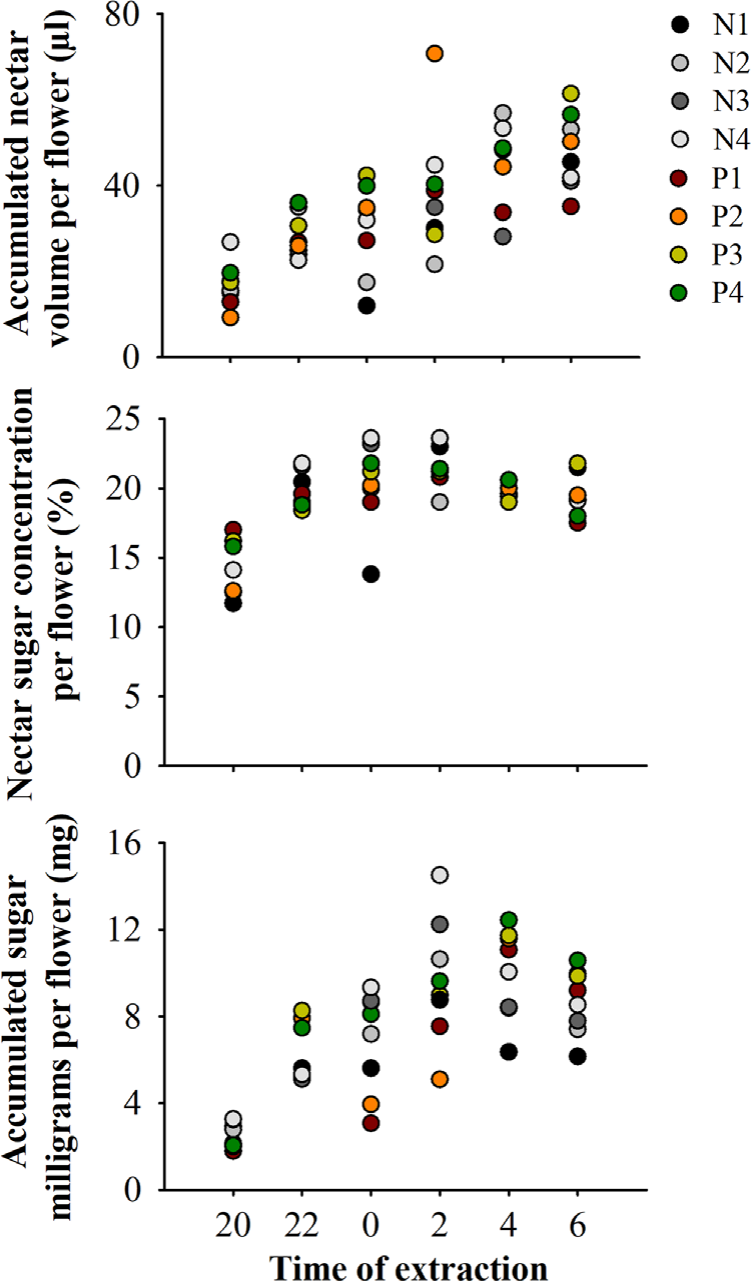
Dynamics of nectar secretion of *Inga vera* at the Brazilian northeastern Atlantic forest, Alagoas state. Nectar secretion pattern in volume (µl), sugar concentration (%) and milligrams (mg) per flower are displayed in natural and planted populations.

**Fig. 4:**
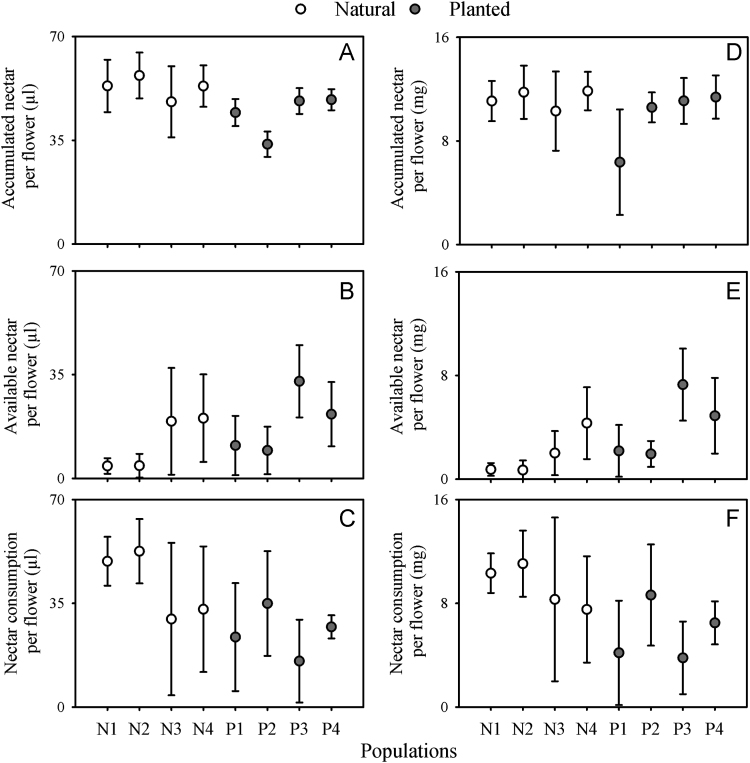
Dynamics of nectar secretion, availability and consumption in natural (A-C) and planted populations (D-F) of *Inga vera* at the Brazilian northeastern Atlantic rain forest, Alagoas state.

Data on the reproduction of *I. vera* across monospecific plantations and natural populations (Fig. 1; Table 1) in the Brazilian northeastern Atlantic forest is shared in this article. Specifically, data on reproductive phenology, nectar secretion and consumption and the reproductive success of *I. vera* are explored. Regarding the reproductive phenophases, similar and moderate synchronization levels of flowering (Fig. 2A) and fruiting (Fig. 2B) are described for planted and natural populations of *I. vera*. In addition, floral nectar, measured by volume (µl), concentration (%) and milligrams of sugars (mg), is similarly secreted during the anthesis across natural and planted populations of *I. vera* (Fig. 3). Reductions on the consumption of floral nectar by pollinators and on the fruit set of monospecific plantations of *I. vera* are documented in Fig. 4 and Table 2, respectively.

**Table 1:** Geographical coordinates of the sampled populations of *Inga vera* (Leguminosae) in the Brazilian northeastern Atlantic forest, Alagoas state.

Population	Latitude	Longitude
Natural		
N1	8°59′46.096′′S	35°50′10.939′′W
N2	8°56′26.277′′S	35°59′42.851′′W
N3	8°58′48.54′′S	36°6′18.746′′W
N4	9°0׳8.543"S	36°7′16.112′′W
Planted		
P1	8°58′37.076′′S	35°54′36.922′′W
P2	8°58′57.916′′S	36°1′41.327′′W
P3	8°58′31.654′′S	36°5′57.268′′W
P4	9°0′16.3′′S	36°6′37.748′′W

**Table 2:** Reproductive success of *Inga vera* (Leguminosae) in natural and planted populations in the Brazilian northeastern Atlantic forest, Alagoas state. Female reproductive success was measured by fruit set (Number of fruits/Number of flowers – Fruit/Flower), seed set (number of seeds per fruit – Seed/Fruit). Male reproductive success was measured by the proportion of pistils with pollen tubes.

		Natural populations				Planted populations			
		N1	N2	N3	N4	P1	P2	P3	P4
Female reproductive success									
Fruit/Flower		0.028	0.023	0.048	0.033	0.019	0.007	0.016	0.023
Seed/Fruit		10.5 ± 3.8	10 ± 3.7	11.17 ± 2.2	11.1 ± 2.5	8.6 ± 2.5	9.0 ± 2.4	10.9 ± 2.6	10.2 ± 3.5
Male reproductive success									
No. of sampled pistils		7	6	8	9	7	8	8	7
No. of pistils with pollen tubes	Style	0	1	0	0	1	0	0	0
	Ovary	3	4	4	4	4	6	4	3
	Total	3	5	4	4	5	6	4	3

## 2 Experimental design, materials, and methods

### 2.1 Flowering and fruiting phenology

The intensity of flowering and fruiting of *I. vera* were monitored monthly along one year for trees located in four natural populations and four monospecific plantations for forest restoration purposes (Fig. 1). In total, the phenology was monitored for 60 individuals of *I. vera*, 30 from natural populations and 30 from planted populations. The number of trees sampled within each planted and natural populations ranged from six to 10. The level of synchronization of an individual in relation to all other sampled individuals (*di*) was calculated to observe changes in flowering and fruiting synchrony between planted and natural populations. We calculated the *di*, which is based on the intensity and number of censuses in which the analyzed phenophase was exhibited, for natural and planted populations. The *di* represents a more accurate measure of the synchrony of a phenophase in relation to other widely used indices, since it considers the intensity and the overlap of the phenophase at the individual or population levels [2].

### 2.2 Floral nectar secretion pattern, availability and consumption

In total, 480 flowers were sampled (240 collected from the four natural populations and 240 from the four planted populations). Amongst the 60 flowers sampled per population, 30 flowers were used in the experiment of nectar secretion pattern and 30 for investigation of nectar availability. Nectar measurements were obtained from flowers of five trees per population. Flowers were classified into six groups, which correspond to the intervals of the nectar extractions. For each group, five flowers were previously bagged (with semipermeable bags) for preventing pollinator visits. In addition, five flowers were kept under natural conditions (exposed to pollinators) to examine the availability of nectar. Bagged flowers represent a measure of nectar accumulated up to the time of extraction, while exposed flowers were accessible to pollinators and, thus, represent a measure of nectar available at the time of extraction. At 20:00 h nectar was extracted from each of the 10 flowers of the first group. At 22:00 h, we did the measurements of nectar on the 10 flowers of the second group and repeated these measurements for the remaining groups of flowers until 6:00 h, which corresponds to the morning after the beginning of the anthesis. Thus, every two hours, we added to our sampling a new measurement of the amount of nectar accumulated and available in the flowers since the beginning of the secretion. The consumption of nectar was calculated by the difference between the amount of nectar accumulated and available in the flowers at the end of the period of active secretion. Nectar volume and sugar concentration were obtained by using microsyringes (Hamilton Microliter^*TM*^ 10 and 25 μl) and a hand-held refractometer (Atago® 0–50%), respectively. Values of milligrams of sugars per flower were obtained by the association between volume and concentration of sugars in the nectar [3].

### 2.3 Reproductive success: pollen tubes, fruit- and seed-set in natural and planted populations

The reproductive success of *I. vera* across planted and natural populations was measured by the fruit set rate and mean number of seeds per fruit, as predictors of the female component, and the proportion of pistils containing pollen tubes, as the male component. Flowers that had finished their anthesis for at least 24 hours were collected to analyze the frequency of occurrence of pollen tubes in their pistils. In order to ensure the growth of pollen tubes up to the ovules, as observed for other *Inga* spp. [3], the interval of 24 hours between the end of the anthesis and the sampling collection was selected. Pistils were fixed in FAA immediately after sampling. In total, 30 pistils from natural populations and 30 from planted populations were sampled. The presence or absence of pollen tubes within the pistils was observed following the fluorescence method [4].

To analyze fruit set under natural conditions, 4953 flowers were observed until the fruit set or flower wilting (2062 flowers for natural populations and 2531 from planted ones). Fruit set was obtained from the ratio between the amount of fruit and flowers previously sampled. To investigate changes in seed production, we collected 60 fruits from natural populations and 60 from planted populations and counted their seeds. Fruits and pistils were collected from seven or eight trees in each reforested area, and from five to eight in each sampled forest remnant. We collected ca. two pistils and two fruits per tree from each population.
